# Simian Foamy Virus Transmission from Apes to Humans, Rural Cameroon

**DOI:** 10.3201/eid1309.061162

**Published:** 2007-09

**Authors:** Sara Calattini, Edouard B.A. Betsem, Alain Froment, Philippe Mauclère, Patricia Tortevoye, Christine Schmitt, Richard Njouom, Ali Saib, Antoine Gessain

**Affiliations:** * Institut Pasteur, Paris, France; † Université de Yaoundé I, Yaoundé, Cameroun; ‡ Centre de l'InstitutdeRecherchepourleDéveloppement, Orléans, France; § Centre Pasteur du Cameroun, Yaoundé, Cameroun; ¶ Hôpital Saint Louis, Paris, France

**Keywords:** Interspecies transmission, retrovirus, foamy, hunters, primates, epidemiology, emerging viruses, research

## Abstract

Simian virus infections of humans are an increasing public health concern. Simian foamy virus (SFV) infections have been reported in persons occupationally exposed to nonhuman primates and in a few hunters in Cameroon. To better understand this retroviral zoonosis in natural settings, we studied persons who lived in southern Cameroon, near nonhuman primate habitats. First we studied a general population of 1,164 adults; 4 were SFV positive according to serologic and molecular assays. Then we studied 85 persons who reported having been bitten or scratched by nonhuman primates; 7/29 (24.1%) of those who had contact with apes (gorillas or chimpanzees) were SFV positive, compared with only 2/56 (3.6%) of those who had had contact with monkeys. These data demonstrate efficient transmission of SFVs to humans in natural settings in central Africa, specifically following ape bites, and viral persistence in the human host.

## Abstract 2

Bites from apes efficiently transmit the foamy virus to humans in natural settings in central Africa.

A large proportion of viral pathogens that have recently emerged in humans have originated in various animals. After initial interspecies transmission, these viruses have evolved and disseminated into the human population through various distinct mechanisms. However, understanding of the initial steps of the emergence of some viruses and associated diseases remains poor. Microbiologic studies of these high-risk populations are thus necessary to obtain new insights into the early events of this emergence process (*1*–*4*).

Nonhuman primates represent a potential source of microbes for humans (*1*,*5*–*12*), e.g., simian immunodeficiency virus and simian T-lymphotropic virus (*12*–*15*). Simian foamy viruses (SFVs) are exogenous complex retroviruses, highly prevalent in several animal species in which they cause persistent infections (*16*–*26*). Phylogenetic analyses have demonstrated a species-specific distribution of such retroviruses. This species specificity indicates a long-term coevolution of SFVs with their natural hosts (*27*), which could explain their possible lack of pathogenicity observed in vivo and the persistence of the infection (*23*,*24*,*28*–*31*). Among nonhuman primate populations, SFV seroprevalence can reach 75%–100% in adults, and SFVs appear to be present at high concentrations in the saliva of infected animals (*16*–*18*,*22*,*29*,*31*).

In humans, SFV infection has been reported in 1%–4% of persons occupationally exposed to nonhuman primates in zoos, primate centers, and laboratories, mainly in North America but also in Europe (*7*–*10*). More recently, naturally acquired SFV infections were described in a few hunters living in Cameroon (*11*) and in 1 person who had had contact with *Macaca fascicularis* in Indonesia (*32*).

After other studies demonstrated high prevalence and genetic diversity of SFVs in monkeys and apes in Gabon and Cameroon (*16*,*17*), we investigated the presence of SFV infection in humans living in these regions. Our goals were to 1) determine, by using specific serologic and molecular methods, the prevalence of SFV infection in the adult population of different ethnic groups (including Pygmies) who lived in rural areas of Cameroon near natural nonhuman primate habitats and who were thus at risk for cross-species transmission; 2) trace the origin of the SFVs infecting these persons by isolation and molecular characterization of the virus; and 3) gain new insights into the epidemiologic determinants and risk factors linked to such naturally acquired retroviral infections, especially the type of nonhuman primates, the circumstances of the contact leading to the infection, and possible intrafamilial transmission of such viruses.

## Materials and Methods

### Populations

**Figure 1:**
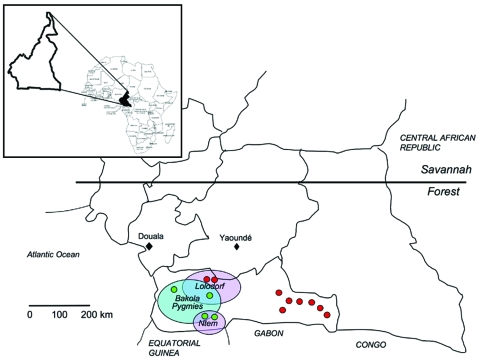
Geographic distribution in Cameroon of the studied populations and the 13 persons infected by simian foamy virus (SFV), according to serologic and molecular results. Red, SFV-positive persons from the hunter study; green, SFV-positive persons from the retrospective study; blue circle, Pygmy area; violet circles, Bantu areas.

The first study, a retrospective study, was based on a large series of samples collected during 1994–2000 for epidemiologic studies on human T-lymphotropic virus (HTLV)-1 and HTLV-2 as well as human herpesvirus 8 (*33*,*34*). The samples originated from adults of 3 ethnic populations: Bakola Pygmies and 2 groups of Bantus, who lived in lowland tropical remote forest areas (Bipindi/Lolodorf and Ntem) in southwestern Cameroon (Figure 1).

The second study, the hunter study, was conducted in 2004–2005 in remote villages near nonhuman primate habitats in the South Province of Cameroon (Figure 1). This study was focused on persons who reported direct and severe contacts (bites, wounds, scratches, other injuries) with animals, especially nonhuman primates, mainly while hunting.

Both studies received clearance from national and local authorities. All participants received detailed information about the study and gave consent. Blood samples were collected in 5–10 mL EDTA tubes. Plasma was available from all participants in the retrospective study, whereas for some in the hunter study, only a few drops of blood were taken by fingerstick and conserved on filter paper (Whatman samples) as described (*35*). See Technical Appendix, for more details.

### Serologic Tests, Virus Isolation, and Molecular Studies

We screened by Western blot (WB) all plasma and Whatman samples for the presence of SFV antibodies as described (*18*,*22*,*26*). Plasma was tested at a 1:100 dilution. For each Whatman sample, a 1-cm punch was diluted in 1 mL of phosphate-buffered saline and tested at a 1:8 dilution (Technical Appendix). Virus isolation, electron microscopy, and immunofluorescence (IFA) were performed as described (*9*,*21*,*26*,*36**;* Technical Appendix).

For the molecular studies, genomic DNA was extracted from the peripheral blood buffy coat by using the QIAamp DNA Blood Mini Kit (QIAGEN, Courtaboeuf, France). Two SFV proviral genomic regions (465 bp of the *integrase* gene and 109 bp of the long terminal repeat [LTR]) were amplified in nested PCR (*18*,*21*,*37*). *Integrase* PCR products were purified, cloned, and sequenced. The GenBank accession numbers of the 13 new *integrase* sequences are DQ838495–DQ838507. Phylogenetic analyses were performed as described (*18*,*38*,*39*; Technical Appendix.)

## Results

### Retrospective Study

The retrospective epidemiologic survey was performed among 1,164 adults (mean age 50.6 years) who lived in the Ocean region of Cameroon (Figure 1; Table 1). The studied populations included 478 Bakola Pygmies (mean age 47.6 years) and 686 Bantus (mean age 52.6 years).

**Table 1:** Serologic results for simian foamy virus retrospective study, rural Cameroon, 1994–2000

Area	Study population						Test results		
	Race	Age range, y	Total no.	Men	Women		Negative	Borderline, no. (%)	Positive, no. (%)
Bipindi Lolodorf	Bakola Pygmies	30–82	478	214	264		448	16 (3.34)	14 (2.92)
	Bantus	40–83	370	180	190		326	40 (10.81)	4 (1.08)
Ntem	Bantus	20–78	316	144	172		283	30 (9.49)	3 (0.9)
Total		20–83	1,164	538	626		1,057	86 (7.38)	21 (1.8)

**Figure 2:**
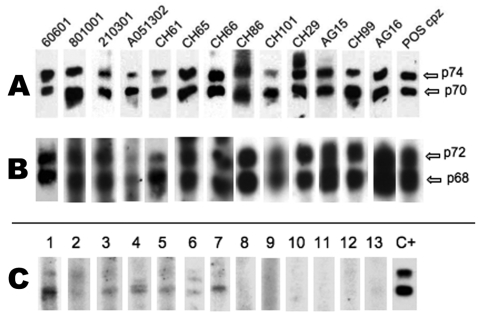
A) Western blot (WB) results based on chimpanzee (cpz) simian foamy virus (SFV) antigens. B) WB results based on monkey simian foamy virus antigens originating from participant AG16. C) Example of sero-indeterminate samples (lanes 1–7) and negative samples (lanes 8–13), detected by cpzSFV WB. Last lane (POS cpz), serum from an SFV-positive chimpanzee.

Of the 1,164 samples tested by WB assay based on chimpanzee foamy virus antigens, 21 (1.8%) were considered clearly positive (strong reactivity to both p70 and p74 ape proteins, Gag doublet) (Figure 2, panel A), 86 (7.4%) were considered borderline/indeterminate (presence of either a faint gag doublet or of at least a strong band of the right size and 1 or few other bands of often low intensity) (Figure 2, panel C), and the remaining 1,057 samples were considered negative (absence of any band) (Figure 2, panel C; Table 1). The 86 indeterminate samples were then tested by WB assay using antigens from a monkey foamy virus (originating from participant AG16, Figure 2, panel B); all were still indeterminate or negative.

DNA was available from 11 of the 21 persons whose WB assay results were positive and from 52 of 86 whose results were borderline/indeterminate. All 63 DNA samples were amplifiable by PCR for β-globin gene. When *integrase* primers were used, PCR was positive for 4 of 63 samples (Table 2). When LTR primers were used, PCR was positive in 3 of these 4 samples (Table 2).

**Table 2:** Demographic and epidemiologic features, serologic and PCR results, 13 SFV-infected inhabitants of rural Cameroon*

Participant code, sex, ethnicity	Year(s) of sample collection	Age at sample collection, at animal contact, y	Type of animal	Wound location	Serologic results (specimen)	LTR/ *Intergase* PCR	*Integrase* sequence	Viral load, copies/500 mg DNA
60601, M, Bantu†‡	1999	67, 30	Gorilla, monkey	Finger	+ (plasma)	+/+	Gorilla	10–100
801001, M, Pygmy†‡§	1996, 1998	60, 35	Gorilla	Leg	+ (plasma)	+/+	Gorilla	100–1,000
210301, M, Pygmy†‡	1996, 2004	68, 35	Gorilla	Leg	+ (plasma)	-/+	Gorilla	1–10
51302, F, Bantu†‡§	1998	40, NK	None?	NK	+ (plasma)	+/+	Chimp.	100–1,000
CH29, M, Pygmy¶	2004, 2005	50, 49	Chimp., gorilla	Finger, foot	+ (DBS, plasma)	+/+	Gorilla	1–10
CH61, M, Bantu*§¶	2004, 2005	65, 52	Gorilla	Hand, arm	+ (plasma)	+/+	Gorilla	10–100
CH65, M, Pygmy¶	2004	58, 26	Gorilla	Head, arm	+ (plasma)	+/+	Gorilla	1–10
CH66, M, Pygmy¶	2004	60, 56	*Cerco.* *nictitans,* Chimp.	Hand, foot	+ (plasma)	+/+	Chimp.	ND
CH86, M, Bantu†¶	2004	62, 47	Gorilla	Hand	+ (plasma)	+/+	Gorilla	1–10
CH99, M, Bantu¶	2004	26, 25	Monkey, species?	Hand	+ (plasma)	–/+	Mandrill	1–10
CH101, M, Bantu¶	2004	76, 65	Gorilla	Hand	+ (plasma)	+/+	Gorilla	1–10
AG15, M, Bantu¶	2004, 2005	71, 28	Chimp.	Hand, foot	+ (DBS, plasma)	+/+	Chimp.	100–1,000
AG16, M, Bantu¶	2004, 2005	43, 23	Monkey *Cerco.*	Foot	+ (DBS, plasma)	–/+	*Cerco.*	1–10

*SFV, simian foamy virus; LTR, long terminal repeat; +, positive; –, negative; DBS, dried blood spot; NK, not known; Chimp., chimpanzee; ND, not determined; *Cerco.,* *Cercopithecus.* The age at contact with nonhuman primate and the type of animal contact concern the results of the field interviews performed for each person found to be infected by SFV. †Wife or husband also tested. ‡Retrospective study participant. §1 (A051302) or 2 (CH61 and 801001) children also tested. ¶Hunter study participant.

**Figure 3:**
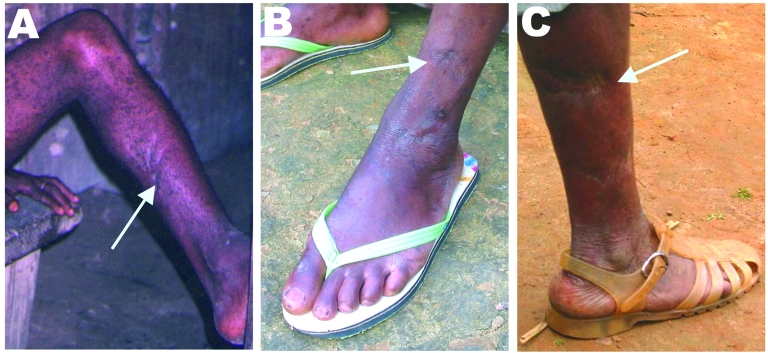
Wounds resulting from bites or scratches from a nonhuman primate. A) Participant no. 801001. B) Participant no. AG16. C) Participant no. 210301.

Field interviews indicated that 3 persons (2 Bakola Pygmies [801001 and 210301] and 1 Bantu [60601]) were frequent hunters and had been severely bitten by gorillas 25–35 years ago (Table 2); all 3 had scars on their legs and fingers (Figure 3). The fourth infected person (A051302) was a Bantu woman who did not recall any bites or injuries from monkeys or apes. However, she had had frequent contact with wild game meat from nonhuman primates while butchering and preparing meals, as is common in this area (*3*,*4*).

Sequence analyses of the 4 *integrase* gene fragments indicated that the 3 persons bitten by gorillas were infected with a gorilla foamy virus. These 3 sequences were similar to the sequence CAM1083 (96.7%–98.5% identity) reported in a Cameroonian hunter infected by a gorilla foamy virus (*11*) and to known sequences of foamy virus from gorillas living in Cameroon and closely related to each other (97%–99% identity). The Bantu woman had been infected by a chimpanzee belonging to *Pan troglodytes troglodytes*, 1 of 2 chimpanzee subspecies endemic to Cameroon (Appendix Figure).

### Hunter Study

Our next step was to not only characterize more cases of such interspecies transmission, looking especially for viral acquisition from other nonhuman primates, but also to assess the frequency of such phenomena and to define the parameters that characterize a risk population. Thus, we focused our work on persons who had regular contact with nonhuman primates, hunters in lowland rain forest regions.

During 2004–2005, field missions were initiated in remote villages of Bantus and Baka Pygmies in different areas of south Cameroon. In each village we specifically asked for persons who had had direct contact and severe bites, scratches, wounds, other injuries from animals, mainly nonhuman primates.

This study included 102 persons, 84 men and 18 women, most of them adults (mean age 40 years, range 2–80 years). Of these 102, 29 (28.4%) had had contact with apes (gorillas, chimpanzees), and 56 (54.9%) with monkeys (*Cercopithecus nictitans),* mandrills, and a few other small monkeys not precisely identified). Thus, 85 of 102 had been in contact with nonhuman primates. Contact with rats, elephants, warthogs, duikers, squirrels, porcupines, and leopards was reported by 17 (16.6%).

From the 102 persons, we obtained 61 plasma samples and 41 dried blood spots. All samples were tested by WB, and 10 (9.7%) were clearly SFV seropositive (Figure 2). Of 15 specimens that were indeterminate/borderline, WB based on monkey FV antigens (originating from participant AG16) showed them all to be negative or indeterminate.

PCR performed on the available DNA (from the 10 WB-seropositive, 8 sero-indeterminate, and 33 seronegative persons) gave positive results for the *integrase* gene in 9 of the 10 WB-positive samples (Table 2) and negative results for the others. The LTR PCR was positive for 7 of 9 *integrase-*positive samples and none of the 42 others.

All 9 SFV-positive persons belonged to the group of 85 persons who had had known contact and bites or scratches from apes or monkeys. Thus, the subsequent epidemiologic analysis was restricted to these 85 (71 men, 14 women; mean age 39 years). According to univariate analysis, foamy virus–positive serologic results were associated with the type of nonhuman primate encountered (monkeys 3.6% vs. apes 24.1%, p = 0.003) and the type of encounter (pets 0% vs. hunting 16.1%, p = 0.022) (Table 3). No other studied risk factor (except age at time of contact) was significantly associated with positive results.

**Table 3:** Univariate analysis results for risk factors for simian foamy virus, 85 persons, rural Cameroon*

Risk factor	Total no. tested	Positive, no. (%)	p value
Age at contact, y			
>45	65	4 (6.2)	
<45	20	5 (25)	0.017
Sex			
Male	71	9 (12.7)	
Female	14	0	0.159
Ethnicity			
Bantu	72	6 (8.3)	
Pygmy	13	3 (23.1)	0.112
Type of animal interaction			
Pet†	29	0	
Hunted	56	9 (16.1)	0.022
Type of nonhuman primate			
Monkey	56	2 (3.6)	
Ape	29	7 (24.1)	0.003
Wound type			
Scratches	9	0	
Bites	76	9 (11.8)	0.275
Wound location			
Upper body	31	2 (6.5 )	
Lower body	54	7 (13)	0.348
Scars			
Absent	12	0	
Present	73	9 (12.3)	0.198

*Only the 85 persons with nown severe bites or scratches from a nonhuman primate. Univariate analyses were performed by using STATA (StataCorp LP, College Station, TX, USA) software with the χ^2^ and Fisher exact tests with critical p value = 0.05. †Most pets were *Cercopithecus nictitans* and mandrills; some were small chimpanzees.

Among the 56 persons who had received severe bites or scratches from nonhuman primates while hunting, 7 (36%) of the 19 that had encountered an ape were infected with SFV, in contrast to only 2 (5.4%) of 37 who had had contact with a small monkey (p<0.05) (data not shown). To determine possible intrafamilial transmission of SFVs, we tested 4 wives and 1 husband of 5 of the index case-participants as well as 5 of their children (Table 2). All were seronegative according to WB.

Of the 9 SFV-positive persons, 7 had been severely bitten by a gorilla (4 persons) or chimpanzee (1 person) 1–53 years ago while hunting (Table 2); some displayed large scars on the legs, arms, feet, or fingers (Figure 3). Hunters CH66 and CH29 had been severely bitten by 2 different animals in 2 separate hunting incidents. The 2 other SFV-positive persons were adult men who had been bitten by a small monkey, including a mandrill and a *C. nictitans* (Table 2).

Phylogenetic analyses of the 9 *integrase* products indicated that all belonged to the large clade of the African SFVs with 5 strains from gorilla, 2 from chimpanzee, 1 from mandrill, and 1 closely related to *Cercopithecus* strains (Appendix Figure). The 2 hunters who had been bitten by 2 different animals were infected with chimpanzee (CH66) and gorilla (CH29) foamy viruses, respectively.

Thus, for each of the 9 case-participants, the match was nearly perfect between the history of contact with a given nonhuman primate species (mainly through severe bites that had occurred decades ago) and the simian virus sequence that was found in the infected person (Table 2).

### In Vivo Virus Persistence

Because each of the 6 persons from whom we obtained 2 samples (plasma, dried blood spots, or both) at different times was SFV positive by WB, persistent infection was evident for each person. The duration of this persistent infection was 1–8 years.

### Isolation of 2 New Foamy Virus Strains

**Figure 4:**
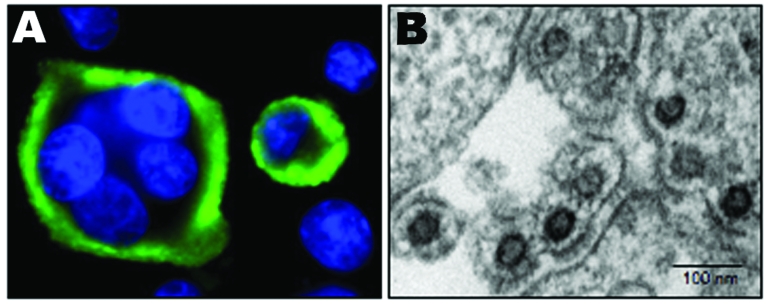
Immunofluorescence and electron microscopy results. A) Typical multinucleated giant cells with a clear seroreactivity of AG16 antigens, determined by using an immunofluorescence assay with positive anti–foamy virus serum, on BHK-21–infected cells cocultivated with stimulated peripheral blood mononuclear cells. B) Electron microscopy of ultrathin sections from cells infected by AG16 foamy virus.

SFV was assayed for 2 persons (AG15 and AG16) from whom blood was available for culture. Giant-cell formation and syncytia were first observed for AG15’s sample after 26 days of coculturing, whereas cytopathic effect (CPE) was detected only after 33 days for AG16’s sample. The destruction of the monolayer of BHK-21 was quite rapid (2–4 days) after the first appearance of the CPE. Syncytia and giant cells showed a strong and clear specific immunofluorescence (Figure 4).

Electron microscopic analyses of cultured cells with a strong CPE demonstrated the presence of multinucleated giant cells. Typical foamy virus particles (diameter 100–110 nm) were frequently observed with several envelope spikes and a spherical central core (Figure 4). Budding of such virus particles was observed, mainly from the membrane surface of the endoplasmic reticulum.

PCR was performed on DNA extracted from the viral isolates after 2 months of culture. Comparative sequence analyses of the *integrase* product showed 100% nucleotide identity for AG16 (*Cercopithecus* strain) and 99.8% for AG15 (chimpanzee strain) between the SFV sequences from the peripheral blood mononuclear cell uncultured DNA and the cultured viral isolate.

### Foamy Virus Load in Buffy Coat

**Figure 5:**
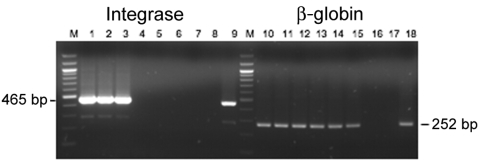
Semiquantitative PCR for *integrase* and β-globin genes using AG15 peripheral blood buffy-coat DNA. Lanes 1–7 and 10–16, serial dilutions of the DNA from 500 ng to 0.5 pg; lanes 8 and 17, negative controls; lanes 9 and 18, positive controls; M, 100-bp ladder.

To determine the peripheral blood viral load in persons infected by SFVs and to check whether the discrepancies in the results between the 2 PCR assays (*integrase* and LTR) could be related to a low viral load (reaching the limits of our PCR sensitivity), we used a semiquantitative PCR assay (*18*). Of the 13 infected persons, 7 (Table 2) had a very low viral load, 1–10 copies in 500 ng of total DNA. For only 4 (all of them positive for both nested PCRs), the viral load was higher, 100–1,000 copies in 500 ng of total DNA (Figure 5; Table 2).

## Discussion

Animal reservoirs are one of the most important sources of emerging infectious diseases that threaten humans. Recent zoonotic transmission of retroviruses has led to the emergence of HIV-1 and HIV-2 in humans (*13*). Nonhuman primates are natural hosts for other retroviruses. Although SFVs have been recently shown to infect persons occupationally exposed to nonhuman primates in zoos and primate centers, little is known about modes of cross-species transmission of these viruses in their natural habitat.

In the current study of adults living in central African regions with high nonhuman primate diversity, ≈2% of 1,164 persons showed clear seroreactivity to SFVs and at least 4 were persistently infected with SFV, with detectable viral sequences in their peripheral leucocyte DNA. These results confirm and extend to other areas of Cameroon the original findings published by Wolfe et al., who found that 10 (1%) of 1,099 of a comparable population had antibodies to SFV with a positive PCR for only 3 of them (*11*). These data, combined with the findings of our hunter study, which identified 9 more SFV-infected persons, demonstrate infection by a large diversity of SFVs in persons from geographically isolated areas. Such retroviral zoonosis is thus widespread and occurs in diverse villages where hunters are frequently in contact with nonhuman primates (*3*,*4*). In another context, a model has predicted that in Bali, Indonesia, for every 1,000 visitors to a monkey temple, approximately 6 will be infected with SFV (*40*).

Our study demonstrates efficient transmission of SFVs to persons in natural settings in central Africa, specifically after the persons had been bitten while hunting, and a viral persistence in the human host. Indeed, >35% of the hunters bitten severely (often with soft tissue crushing, tearing, and bleeding) by a gorilla or a chimpanzee were SFV infected. This strongly suggests that in a natural situation, contact of human blood with the saliva of an adult ape or monkey is the key factor for SFV transmission to humans. This situation is similar to that of persons occupationally exposed to nonhuman primates in zoos and primate centers, as nearly all of them reported having been bitten by monkeys or apes (*5*,*9*,*10*). Some studies have shown that SFVs are present at high concentration in the saliva (with viral replication) of infected animals (*29*,*31*). We recently provided evidence that *Macaca tonkeana* mostly acquire SFVs through severe bites, mainly young adults when they compete for sex partners (*18*). In our study, contact with pets was not found to be associated with SFV infection. This might be because pet bites mainly cause superficial tissue damage and rarely cause serious wounds and because some of the animals are probably not SFV infected due to their young age when captured.

In our study, SFV *integrase* or LTR sequences were not detected in several of the persons who were confirmed seropositive by WB. Although the presence of divergent SFVs could explain such discrepancy, low viral load in the blood samples is more likely, because our PCR primers have been shown to amplify a large variety of African SFVs but also several rather divergent Asian SFVs (*16*–*18*,*22*). This lack of detection of FV sequences by PCR may also indicate nonspecific reactivity with SFV Gag antigens. Lack of SFV sequences has also been recently reported in the peripheral blood mononuclear cell DNA of 7 of 10 African hunters who were SFV seropositive according to WB (*11*).

We provide the first data, to our knowledge, on the quantification of viral load of SFVs in humans. Our results, based on 13 infected persons, indicate a low viral load in most persons but a large range (1–1,000 copies in 500 ng of total peripheral blood leukocytes DNA). These viral loads are comparable to those in wild-born chimpanzees (*16*) and captive *M. tonkeana* (*18*).

Our work did not demonstrate the presence of SFV in the spouses of 5 index case-participants and in 5 of their children. Combined with the scarce published findings on this topic, these results suggest that SFV transmission among humans does not occur easily by sexual contact or saliva exposure (*8*–*10*).

Another concern is the illness and death that might be associated with these retroviral persistent infections after interspecies transmission. The apparent lack of pathogenicity of SFV infection in humans, which is still based on a limited number of cases, contrasts strongly with the massive in vitro lytic properties of these viruses in monkey and human cells (*8*–*10*,*18*,*30*). The selection bias inherent in the enrollment of healthy persons in our study, as well as in those enrolled by Wolfe et al. (*11*) and Switzer et al. (*10*), greatly limits the ability to identify any potential acute or severe associated diseases. A case-control study based on a larger number of SFV-infected persons would help shed light on possible chronic diseases or biological abnormalities associated with human SFV infection. SFV infection in immunocompromised persons, especially those with HIV infection, could also heighten public health concerns because such coinfection is probable in central African areas where HIV-1 is highly endemic.

## Supplementary Material

Technical Appendix Ethnographic and field survey information

Appendix Figure Phylogenetic tree generated on a 425-bp fragment of the integrase simian foamy virus (SFV) gene. The 13 new SFV sequences described in this study are shown in red. Numbers at each node indicate the percentage of bootstrap samples (1,000 replicates); only values >60% are shown. The branch lengths are drawn to scale with the bar indicating 0.1-nt replacement per site. The tree was rooted by using the Asian Macaca mulatta (MmuSFVmac) sequence.
