# Supplementary material for: Simian Foamy Virus Transmission from Apes to Humans, Rural Cameroon
Source: Emerg Infect Dis. 2007 Sep;13(9):1314–20. doi: 10.3201/eid1309.061162 (PMC2857270; doi:10.3201/eid1309.061162)
Supplement: Technical Appendix — Ethnographic and field survey information [file 06-1162_Techapp-s1.pdf]

Publisher: CDC; Journal: Emerging Infectious Diseases
Article Type: Research; Volume: 13; Issue: 9; Year: 2007; Article ID: 06-1162
DOI: 10.3201/eid1309.061162; TOC Head: Research

Technical Appendix

1. Ethnographic and field survey information

The Bakola Pygmies (also called Baguelli) are an ethnic group comprising about 4.000/5.000 individuals, living mainly in the Ocean Department of southern Cameroon. This group, traditionally hunter-gatherers, is scattered throughout their forest territories for hunting; they now practice rudimentary agriculture but also exchange game for staple foods and manufactured goods with neighboring Bantu farmers. Between 1994 and 2000, several settlements of Bakola Pygmies and of villages of Bantus (mainly of the Ngumba, Fang, Ntumu, Mvaie, and Bulu tribes) living in their very close neighborhood were visited. The survey was done after authorization of the local authorities (villages and settlements chiefs) with information to each participant.

The Baka Pygmies are an ethnic group comprising in Cameroon about 25.000/30.000 individuals, living mainly in the South and East Departments of Southern Cameroon. This group has conserved a quite traditional way of life and the Baka are the least admixed Pygmies living in Cameroon. The “hunters study” was mainly performed in this group as well as in the Bantus groups living in their neighborhood as the Fang, Bulu, Ewondo and Ngumba tribes. The survey, carried out in 2004-2005, was done after authorization of national (Ministry of Health, and National Ethic Committee of Cameroon) and local authorities (villages and settlements chiefs) with information to each participant.

For both studies, Informed consent was obtained from all adults and parents of minors included in the study. Furthermore, all of the participants underwent a medical examination and were treated if necessary according to the local medical facilities. A 5 or 10 ml blood sample was taken on EDTA tubes.

2. Methods

Serological tests

The Western blot (WB) assay was performed using, as a source of foamy viral antigens, a cellular lysate of BHK-21 cell line infected with a chimpanzee SFV strain. All of the

indeterminate samples were then screened with another WB assay using, as viral antigen, a cellular lysate of BHK-21 cell line infected with a Cercopithecus SFV strain isolated from the AG16 case. For both tests, plasma samples were tested at a 1:100 dilution. Concerning the dried blood spot ("whatman samples"), a punch of approximately 1 cm in diameter was diluted in 1 ml of PBS and tested at a 1:8 dilution. WB seropositivity for foamy viruses was defined as the presence of a clear reactivity to the Gag doublet of 70 and 74 KDa for the Cpz assay and of 68KDa and 72 KDa for the monkey assay.

Virus isolation

Virus isolation was performed in two samples (AG15 and AG16) showing strong WB seropositivity, as previously described (14, 34, 37, 50). Briefly, BHK-21 cells were maintained in DMEM medium supplemented with 5% fetal calf serum (FCS) and antibiotics. Fresh blood samples were collected in EDTA tubes and PBMCs (Peripheral Blood Mononuclear Cells) were isolated on a Ficoll-Hypaque gradient. PBMCs were then maintained for 2 days in RPMI medium containing 20% FCS, antibiotics and phytohemagglutinin (PHA) at 3 μg/ml or Protein A at 0,1 μg/ml and further stimulated with IL-2 (100U/ml). After 2 days of stimulation, PBMCs were co-cultivated with BHK-21 cells. Cultures were checked daily for syncytial cytopathic effect (CPE) typical of FV infection.

For transmission electron microscopy, cells were fixed in 2.5% glutaraldehyde and 1% paraformaldehyde in 0.15 M cacodylate buffer complemented with $MgCl_2$, $CaCl_2$ and sucrose at 0.1M. After 2 days at 4°C, the filters were washed during 2 hours in cacodylate buffer and treated with a 1% osmium teroxide solution and 1% potassium ferrocyanide for 1 hour at room temperature. Cells were dehydrated in ethanol and embedded in epoxy resin at 60°C for 48hrs. Ultrathin sections were obtained on a microtome Leica ultracut UCT. Sections were then examined in a Jeol 1200 EX electron microscope.

Indirect immunofluorescence

An indirect IF assay was performed on co-cultivated cells at 35 days post-infection. For the antibody positive control, we used, as the primary antibody, a serum derived from rabbit experimentally infected with a chimpanzee SFV strain; the secondary antibody was fluorescein-conjugated goat anti-rabbit diluted at 1:500. Cells were then mounted with DAPI-containing mounting medium and visualized with a Zeiss Axioplan 2 imaging microscope X40 using a Zeiss

Publisher: CDC; Journal: Emerging Infectious Diseases
Article Type: Research; Volume: 13; Issue: 9; Year: 2007; Article ID: 06-1162
DOI: 10.3201/eid1309.061162; TOC Head: Research

Axiocam Hrc (color) camera and Zeiss Apotome software. For each reaction, a negative and positive cell control was added. The positive controls were BHK-21 cells infected with a chimpanzee SFV strain, while the negative controls consisted of uninfected BHK-21 cells.

Molecular studies

High molecular weight genomic DNA was extracted from the peripheral blood buffy-coat of 114 samples using the Qiagen kit (QIAmp blood Mini Kit, Courtaboeuf, France). These 114 samples correspond to 63 individuals (11 positive and 52 indeterminate WB) from the retrospective study and 51 (10 positive, 8 indeterminate and 33 negative WB) from the hunters study.

Two SFV proviral genomic regions (a 465 bp fragment of the *integrase* gene and a 109 bp fragment of the LTR) were amplified using two nested PCR (for primer sequences see ref 21, 37). The presence and quality of the extracted DNA were verified by amplification of a ß-globin gene fragment.

To estimate the viral load, DNA samples showing a PCR positive result were then amplified with a semi-quantitative assay. For this purpose, six 10-fold serial dilutions of the DNA ranging from 500 ng to 0.5 pg were amplified under the same conditions than the first qualitative PCR; at the same time, each dilution was amplified separately for a ß-globin gene fragment.

Integrase PCR products were purified, cloned in a vector, and sequenced using the BigDye terminator cycle kit and an ABI 3100 automated sequencer (Applied Biosystem).

To determine the sensibility of our PCR assay, DNA from a cell line (HFV-2) containing 2 copies of integrated DNA/cell was extracted and measured by spectrophotometer. Serial dilutions of this DNA ranging from 1 μg (i.e. 150000 cells) to 0,01 ng were amplified in nested PCR for the *integrase* gene and in simple PCR (50 cycles) for the ß globin gene (Figure 1 supp., lanes 1-7). For the *integrase* PCR, each dilution was diluted in 100 ng of SFV negative DNA; the last positive dilution for *integrase* PCR (lane 7) corresponds to 1,5 cells or 3 copies of HFV. The last positive dilution for the ß globin PCR (lane 7) corresponds to 1,5 cells or 3 copies of ß globin. It means that the sensibility of our PCR ranges between 1-10 copies.

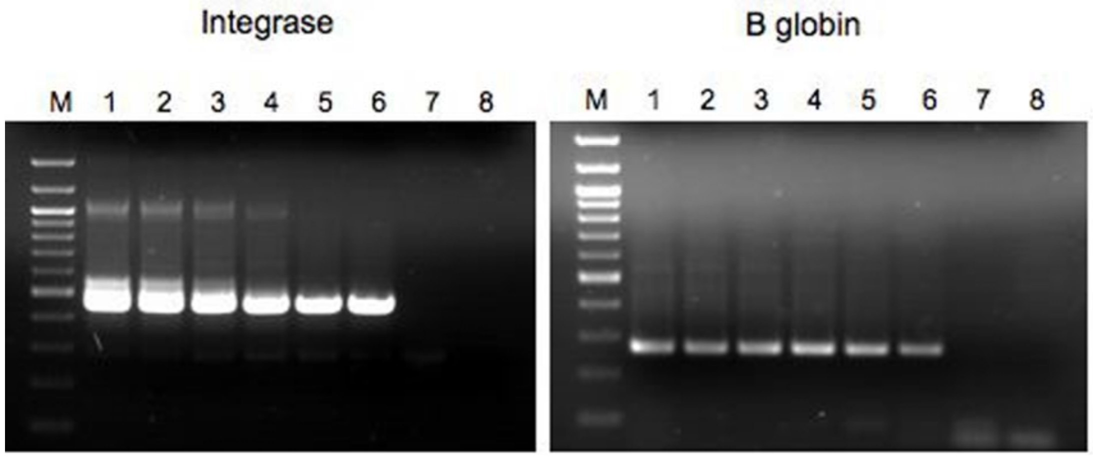

Phylogenetic analyses

Multiple nucleotide sequence alignments were performed with the DAMBE program on the basis of a previous amino-acid alignment created from the original sequences. The final alignment was submitted to the Model Test program to select the best phylogenetical model to apply for the phylogenetical analyses. The best phylogenetical model, selected using the Model Test was the TVM+I+G model (–lnL = 8546,7) with a shape of 0,8933 and a pinvar of 0,3254. The phylogeny was derived by the Neighbour-Joining method (with a bootstrap value of 1000), performed in Paup program.
