# Supplementary figures and images for: Simian Foamy Virus Transmission from Apes to Humans, Rural Cameroon
Source: Emerg Infect Dis. 2007 Sep;13(9):1314–20. doi: 10.3201/eid1309.061162 (PMC2857270; doi:10.3201/eid1309.061162)

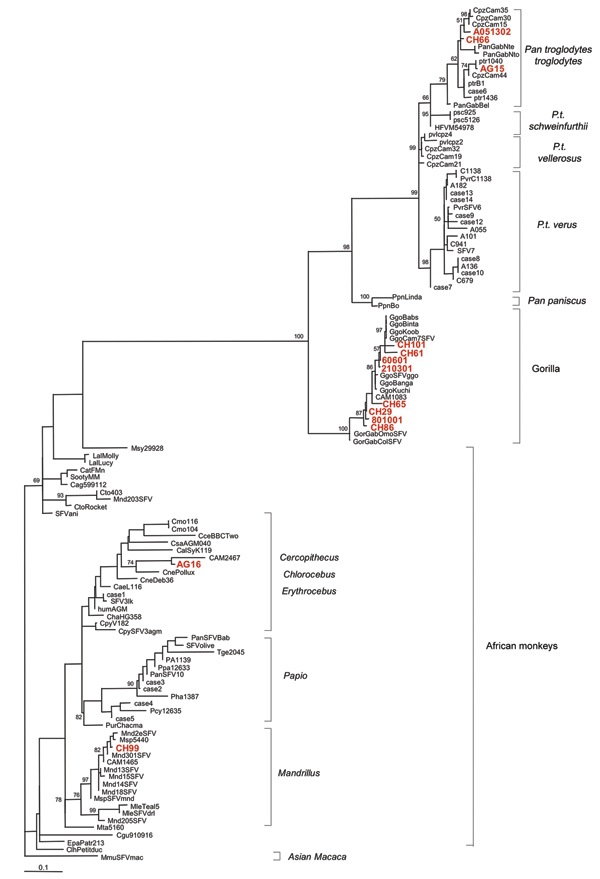

Supplement: Appendix Figure — Phylogenetic tree generated on a 425-bp fragment of the integrase simian foamy virus (SFV) gene. The 13 new SFV sequences described in this study are shown in red. Numbers at each node indicate the percentage of bootstrap samples (1,000 replicates); only values >60% are shown. The branch lengths are drawn to scale with the bar indicating 0.1-nt replacement per site. The tree was rooted by using the Asian Macaca mulatta (MmuSFVmac) sequence. [file 06-1162_appF-s2.gif]
